# Association of Sleep Duration With Intracranial Atherosclerosis and Cerebral Small Vessel Disease: A Mediation by Metabolic Factors

**DOI:** 10.1002/cns.70797

**Published:** 2026-02-17

**Authors:** Hongbin Chen, Anqi Zhang, Weiqi Chen, Xueli Cai, Mengyuan Zhou, Shan Li, Jing Jing, Tiemin Wei, Yongjun Wang, Yuesong Pan, Yilong Wang

**Affiliations:** ^1^ Department of Neurology, Beijing Tiantan Hospital Capital Medical University Beijing China; ^2^ Department of Neurology Fujian Medical University Union Hospital Fuzhou China; ^3^ China National Clinical Research Center for Neurological Diseases Beijing China; ^4^ Department of Neurology The Fifth Affiliated Hospital of Wenzhou Medical University Zhejiang China; ^5^ Lishui Clinical Research Center for Neurological Diseases Zhejiang China; ^6^ Cerebrovascular Research Lab The Fifth Affiliated Hospital of Wenzhou Medical University Zhejiang China; ^7^ Department of Cardiology The Fifth Affiliated Hospital of Wenzhou Medical University Zhejiang China; ^8^ Chinese Institute for Brain Research Beijing China; ^9^ National Center for Neurological Disorders Beijing China; ^10^ Advanced Innovation Center for Human Brain Protection Capital Medical University Beijing China; ^11^ Beijing Laboratory of Oral Health Capital Medical University Beijing China; ^12^ Laboratory for Clinical Medicine Capital Medical University Beijing China

**Keywords:** cerebral small vessel disease, intracranial atherosclerosis, mediation, metabolic factors, sleep duration

## Abstract

**Aims:**

This study aimed to explore the relationship between sleep duration and intracranial atherosclerosis and cerebral small vessel disease (CSVD), and pinpoint the potential mediating factors.

**Methods:**

Data were derived from the cross‐sectional baseline survey of the PRECISE (Poly‐vascular Evaluation for Cognitive Impairment and Vascular Events) study. Participants were divided into short sleep (< 7 h), normal sleep (7–9 h), and long sleep (> 9 h) groups. The associations of sleep duration with intracranial atherosclerotic plaque, intracranial atherosclerotic burden, total CSVD score, and CSVD imaging markers were evaluated.

**Results:**

We enrolled 3038 participants (53.5% women; mean age: 61.2 ± 6.7 years). A long sleep duration was correlated with a higher risk of intracranial atherosclerotic plaque (long vs. normal: odds ratio [OR], 1.40 [95% CI, 1.10–1.78]), intracranial atherosclerotic burden (long vs. normal: common OR, 1.38 [95% CI, 1.09–1.75]), enlarged perivascular space in the basal ganglia (BG‐EPVS) (long vs. normal: OR, 1.45 [95% CI, 1.07–1.97]); a short sleep duration indicated a rise in lacune (short vs. normal: OR, 1.67 [95% CI, 1.06–2.63]) after adjustment for covariates. The association of a long sleep duration with intracranial atherosclerotic plaque, intracranial atherosclerotic burden, and BG‐EPVS (mediation percentage: 35.4%, 34.0%, 20.3%, respectively) was partially mediated by metabolic factors of blood pressure and fasting plasma glucose.

**Conclusion:**

Aberrant sleep duration may increase the potential risk for intracranial atherosclerosis and CSVD, which can be partially mediated by blood pressure and fasting plasma glucose. These findings highlight the benefits of clinical management of metabolic factors for those with aberrant sleep duration to prevent intracranial atherosclerosis and CSVD.

**Trial Registration:**

NCT03178448

## Graphical abstract

Data of community dweller adults were derived from a survey study (3038 participants). We found that sleep duration is associated with intracranial atherosclerosis and CSVD, and the association of long sleep duration with intracranial atherosclerosis and CSVD was partially mediated by blood pressure and fasting plasma glucose.

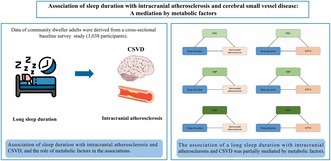

## 1 Introduction

Sleep is crucial for human health, with individuals dedicating approximately one‐third of their time to sleep [1]. Current research demonstrates that a long or short sleep duration can heighten the risks of stroke, cardiovascular diseases, heart disease, angina, and heart attacks [2]. Therefore, commonplace as it is, sleep may be implicated in the pathogenesis of cerebrovascular diseases.

Mechanistically, sleep regulates the secretion of hypocretin and chronic sleep fragmentation can trigger the loss of hypocretin and aggravate atherosclerosis [3]. Recent studies suggest that a long or short sleep duration may exacerbate carotid atherosclerosis [4, 5]. Other studies indicate that patients with ≥ 9 h of sleep may experience an increase in white matter hyperintensity volume [6]. However, available literature has largely failed to present the total cerebral small vessel disease (CSVD) scores and a complete evaluation of imaging markers for intracranial atherosclerosis and CSVD. The role of sleep in the pathogenesis of cerebrovascular diseases, such as CSVD and intracranial atherosclerosis, and the underlying mechanism has been squarely left unexplored.

Functionally, sleep duration can impact several metabolic factors such as blood glucose, lipids, and blood pressure [7, 8, 9]. Meanwhile, these metabolic factors are closely associated with intracranial atherosclerosis and CSVD [10, 11, 12]. Therefore, we speculate that sleep may affect these intracranial vascular anomalies via metabolic factors.

In this study, data were derived from a community‐based study to explore the relationship between sleep duration and intracranial atherosclerosis and CSVD and pinpoint the potential mediating factors. The findings may extend our understanding of the involvement of sleep in the pathogenesis of cerebrovascular diseases and atherosclerosis, and provide clinical evidence for the management of intracranial atherosclerosis and CSVD.

## 2 Methods

### 2.1 Participants

Data were retrieved from the baseline survey of the PRECISE (Poly‐vascular Evaluation for Cognitive Impairment and Vascular Events) study (NCT03178448), which is a prospective cohort, with 3067 elderly participants (aged 50 to 75 years) from a community of six villages and four neighborhoods in Lishui City, China. The theoretical basis, design, and baseline characteristics of the study have been published elsewhere [13]. This study employed cluster sampling from living communities (rather than occupational communities), which enabled the demographic characteristics and medical histories of the participants to match the national survey data closely. This group representativeness allowed the study to accurately assess the prevalence of clinical or subclinical multi‐vessel disease and ensured that the research results were universally applicable, suitable for the general population [13]. The study utilized advanced vascular imaging techniques for a comprehensive assessment of intracranial and extracranial arterial stenosis and plaque in these subjects.

Participants meeting the following criteria were excluded from the study: (1) patients with contraindications to magnetic resonance imaging (MRI) or computed tomography angiography (CTA); (2) those with a life expectancy of ≤ 4 years due to advanced cancer or other serious illnesses; (3) individuals with psychiatric disorders; and (4) participants with obstructive sleep apnea or without intra‐/extra‐cranial atherosclerosis imaging, CSVD imaging, or sleep data.

### 2.2 Ethics Statement

The current study protocol was reviewed by the ethical committees of Lishui Hospital (IRB approval number: 2016‐42) and Beijing Tiantan Hospital (IRB approval number: KY2017‐010‐01). The informed consent was obtained from all participants before enrollment.

### 2.3 Clinical and Biochemical Assessment

The baseline data were collected at Lishui Hospital with standardized questionnaires by trained researchers. Demographic details, concomitant medications, medical history, and behavioral scales (sleep, smoking, drinking, and body mass index [BMI]) were obtained during face‐to‐face interviews, in which data on previously diagnosed obstructive sleep apnea were also recorded. Samples of venous blood were obtained the next morning after a fasting of ≥ 8 h. Fasting plasma glucose (FPG), total cholesterol (TC), triglycerides (TG), high‐density lipoprotein cholesterol (HDL‐C), low‐density lipoprotein cholesterol (LDL‐C), and other biomarkers were measured following standardized procedures.

### 2.4 Measurement of Sleep Duration

Self‐reported sleep duration per night over the past month was collected according to face‐to‐face interviews at baseline. Accordingly, the participants were then categorized into 3 groups: long sleep (> 9 h), normal sleep (7–9 h), and short sleep (< 7 h) [14].

### 2.5 Measurement of Intra‐ and Extra‐Cranial Atherosclerosis

Intra/extracranial atherosclerotic plaques and stenoses were assessed by MRI by trained investigators on a 3.0T scanner (scanner: Ingenia 3.0T [Philips]). The MRI sequences consisted of intraplaque hemorrhage imaging, simultaneous non‐contrast angiography, 3‐dimensional anisotropic high‐resolution black‐blooded T1w vessel‐wall imaging, and 3‐dimensional time‐of‐flight magnetic resonance angiography (3‐D‐TOF MRA). MRI data were collected on the discs in a DICOM format and examined by 2 radiologists (Dongxiao Yao and Huihui Liu) at the imaging research center of Beijing Tiantan Hospital. The rating inconsistencies were resolved by another senior neurologist (Jing Jing) The inter‐rater reliability for the presence of plaques and artery stenosis (≥ 50%) in intra‐ and extra‐cranial arteries was detected by the Cohens *κ* (intracranial artery: Cohen *κ* = 0.97 and 0.79; extracranial artery: Cohen *κ* = 0.94 and 0.86).

The atherosclerotic plaque was designated as eccentric wall thickening observed on 3‐D‐TOF MRA or black blood MR images, regardless of luminal narrowing [15]. The atherosclerotic status was determined with a semi‐quantitative score according to the degree of atherosclerotic plaque and stenosis (0, no atherosclerotic plaque; (1) atherosclerotic plaque without significant luminal stenosis of < 50%; (2) stenosis of 50%–69%; (3) stenosis of 70%–99%; (4) occlusion) [16]. The assessed intracranial arterial segments involved basal, anterior cerebral arteries (A1, A2), bilateral internal carotid arteries, posterior cerebral arteries (P1, P2), middle cerebral arteries (M1, M2), and vertebral arteries (V4). The extracranial arterial segments included the proximal internal carotid artery, the common carotid artery, and the vertebral arteries (V1, V2, V3). The intracranial or extracranial atherosclerotic scores in each arterial segment were respectively summed to calculate the intracranial or extracranial atherosclerotic burden, which were graded as 0, 1, 2–3, and ≥ 4 scores [16].

### 2.6 Measurement of CSVD

All MRI scans were further analyzed according to standardized protocols by trained researchers. The MRI markers of CSVD were defined according to the Standards for Reporting Vascular Changes on Neuroimaging Criteria [17]. The white matter hyperintensity (WMH) indicated a heightened brightness in the region of white matter on T2‐weighted MRI scans. The severity of periventricular and deep WMH was assessed using the Fazekas rating scale [18]. Lacunes were defined as round or ovoid lesions with cerebrospinal fluid signal, measured between 3 and 20 mm in diameter. Cerebral microbleeds (CMBs) appeared as round, hypodense lesions measured from 2 to 10 mm on gradient‐recalled echo or susceptibility‐weighted images. The total count of lacunes and CMBs was recorded. Enlarged perivascular spaces in the basal ganglia (BG‐EPVS) were defined as small (< 3 mm) punctate or linear hyperintensities on T2‐weighted images. The perivascular spaces (PVS) in the basal ganglia were assessed using the semi‐quantitative rating scale developed by the Edinburgh group [19]. Each imaging marker of CSVD was evaluated by 4 trained raters (M. Zhou, Y. Chen, J. Pi, and M. Zhao), with each marker independently rated by two raters, who were blinded to the clinical data. Inconsistencies were reviewed by a senior neurologist (Y.Y.), also blinded to initial assessments. The inter‐rater reliability, measured by κ‐coefficients, was 0.80 for lacune presence, 0.82 for the Fazekas WMH scale, 0.90 for PVS severity, and 0.80 for CMB presence, indicating a satisfactory rating consistency.

The overall CSVD scores were assessed using an ordinal scale ranging from 0 to 4. As previously reported [20], one point was assigned for the presence of periventricular WMH (Fazekas 3) or deep WMH (Fazekas 2–3), lacunes, cerebral microbleeds (CMBs), and moderate‐to‐severe basal ganglia PVS (*n* = 10–20). Additionally, according to the recently‐validated modified CSVD scores [21], ranging from 0 to 6, one point was allocated for lacunes, CMB burden (*n* = 1–4), severe basal ganglia PVS (*n* > 20), and modified WMH burden (total periventricular + subcortical WMH grade 3–4); two points were assigned for CMB burden (*n* ≥ 5) and modified WMH burden (total periventricular + subcortical WMH grade 5–6).

### 2.7 Statistical Analysis

Continuous variables were expressed as mean ± standard deviation (SD) or median [interquartile range (IQR)] and examined by ANOVA or the Kruskal–Wallis test. Categorical variables were depicted as frequency (%) and assessed by the Chi‐squared test or Fisher's exact test.

The associations of sleep duration with the presence of atherosclerotic plaque, CSVD imaging markers, and presence of CSVD were evaluated by binary logistic regression. Its correlations with total CSVD score, atherosclerotic burden, and modified total CSVD score were examined by ordinal logistic regression. The odds ratio (OR) or common odds ratio (cOR) with 95% confidence intervals (CI) were computed. Our selection of covariates was based on clinical relevance and previous studies [4, 5]. In all models, adjustment was performed for sex, age, body mass index (BMI), current smoking, and current drinking.

We developed a simple mediation model to analyze the effect of metabolic factors on the correlation of sleep duration with intracranial atherosclerosis and CSVD. In this context, the overall association between sleep duration and intracranial atherosclerosis and CSVD was divided into two distinct components: natural indirect (or mediated) and natural direct effects [22]. The former indicates the effect of sleep duration on intracranial atherosclerosis and CSVD through metabolic factors, whereas the latter suggests pathways that do not involve these metrics. To demonstrate the reliability of the mediation analysis, three pathways needed to be identified: the association of sleep duration with large and small vascular lesions; that of sleep duration with metabolic factors; and that of metabolic factors with intracranial atherosclerosis and CSVD. The proportion mediated by metabolic factors was computed by dividing the value of natural indirect effect (mediated effect) by that of the total effect [22, 23].

All analyses were conducted with SAS 9.4 (SAS Institute Inc., Cary, NC, USA) and R 4.2.2 (R Development Core Team). A two‐sided *p* < 0.05 was deemed statistically significant.

## 3 Results

### 3.1 Baseline Characteristics of the Participants

A total of 3067 participants were included in the PRECISE study. After the exclusion of 20 participants for a lack of intracranial or extracranial atherosclerosis images, 4 participants for absence of MR images, and 5 for obstructive sleep apnea, 3038 qualified subjects were enrolled, with a mean age of 61.2 ± 6.7 years and females accounting for 53.5% (Figure S1). The baseline demographic details of the 3 sleep duration groups are summarized in Table 1. The long sleep duration group was much older and featured a higher level of diastolic blood pressure (DBP), systolic blood pressure (SBP), FPG, and HDL‐C.

**TABLE 1:** Baseline characteristics according to sleep duration.

Variable	Total (*n* = 3038)	Sleep duration^a^			*p*
		Short (*n* = 361)	Normal (*n* = 2167)	Long (*n* = 510)	
Demographic data					
Age, years, mean ± SD	61.2 ± 6.7	60.6 ± 6.5	61.0 ± 6.6	62.4 ± 7.0	< 0.001
Female sex, *n* (%)	1626 (53.5)	209 (57.9)	1165 (53.8)	252 (49.4)	0.04
BMI, kg/m^2^, mean ± SD	23.8 ± 3.0	24.1 ± 3.1	23.7 ± 3.0	23.8 ± 3.2	0.045
Current smoking, *n* (%)	622 (20.5)	72 (19.9)	442 (20.4)	108 (21.2)	0.89
Current drinking, *n* (%)	572 (18.8)	63 (17.5)	402 (18.6)	107 (21.0)	0.35
Medical history, *n* (%)					
Hypertension	1301 (42.8)	152 (42.1)	913 (42.1)	236 (46.3)	0.23
Diabetes	655 (21.6)	88 (24.4)	448 (20.7)	119 (23.3)	0.16
Dyslipidemia	1267 (41.7)	167 (46.3)	905 (41.8)	195 (38.2)	0.06
Educational level, *n* (%)					
Illiteracy	559 (18.4)	56 (15.5)	355 (16.4)	148 (29.0)	< 0.001
Primary school	793 (26.1)	82 (22.7)	536 (24.7)	175 (34.3)	
Junior high school	922 (30.3)	117 (32.4)	680 (31.4)	125 (24.5)	
Senior high school	576 (19.0)	74 (20.5)	449 (20.7)	53 (10.4)	
Graduate and above	188 (6.2)	32 (8.9)	147 (6.8)	9 (1.8)	
SBP, mmHg, mean ± SD	129.2 ± 16.3	127.8 ± 16.2	128.7 ± 16.0	132.6 ± 17.4	< 0.001
DBP, mmHg, mean ± SD	75.2 ± 9.0	74.4 ± 8.8	75.0 ± 9.0	76.6 ± 9.3	< 0.001
Fasting plasma glucose, mmol/L, median (IQR)	5.6 (5.2–6.1)	5.6 (5.2–6.2)	5.6 (5.2–6.1)	5.7 (5.3–6.3)	0.001
TC, mmol/L, median (IQR)	5.2 (4.6–5.9)	5.2 (4.6–6.0)	5.2 (4.6–5.9)	5.3 (4.7–5.9)	0.60
LDL‐C, mmol/L, median (IQR)	2.7 (2.3–3.3)	2.8 (2.3–3.4)	2.7 (2.3–3.2)	2.8 (2.2–3.3)	0.17
HDL‐C, mmol/L, median (IQR)	1.3 (1.1–1.6)	1.3 (1.1–1.5)	1.3 (1.1–1.6)	1.4 (1.2–1.6)	0.02
eGFR, mL/min/1.73 m^2^, median (IQR)	94.9 (87.4–100.3)	95.3 (89.2–100.3)	94.9 (87.4–100.4)	94.4 (86.3–100.0)	0.28
Medication use, *n* (%)					
Antihypertensive	810 (26.7)	94 (26.0)	583 (26.9)	133 (26.1)	0.89
Lipid‐lowing	115 (3.8)	16 (4.4)	83 (3.8)	16 (3.1)	0.60
Antiplatelet	78 (2.6)	10 (2.8)	54 (2.5)	14 (2.8)	0.92
Antidiabetic	273 (9.0)	31 (8.6)	193 (8.9)	49 (9.6)	0.85

Abbreviations: BMI, body mass index; DBP, diastolic blood pressure; eGFR, estimated glomerular filtration rate; FPG, fasting plasma glucose; HDL‐C, high‐density lipoprotein cholesterol; LDL‐C, low‐density lipoprotein cholesterol; SBP, systolic blood pressure; SD, standard deviation; TC, total cholesterol.

^a^ The degree of sleep duration was graded as short sleep (< 7 h), normal sleep (7–9 h), and long sleep (> 9 h).

### 3.2 Association of Sleep Duration With Intracranial Atherosclerosis and CSVD

**FIGURE 1:**
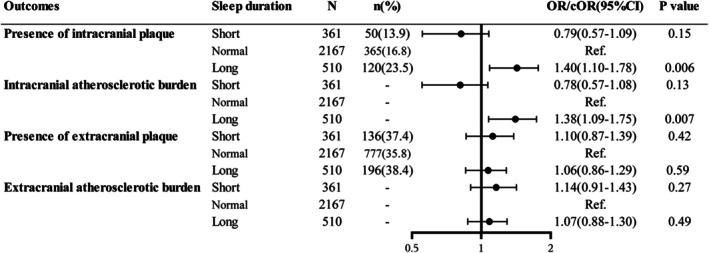
Forest plots for the association of sleep duration with intracranial and extracranial atherosclerotic plaque and burden. CI, confidence interval; cOR, common odds ratio; OR, odds ratio. The degree of sleep duration was graded as short sleep (< 7 h), normal sleep (7–9 h), and long sleep (> 9 h). The degree of intracranial atherosclerotic burden was graded as scores of 0, 1, 2–3, and ≥ 4. The degree of extracranial atherosclerotic burden was graded as scores of 0, 1, 2–3, and ≥ 4. All models were adjusted for age, sex, body mass index, current smoking, and current drinking. ORs were estimated for the presence of intracranial and extracranial plaque; cORs were used for intracranial and extracranial atherosclerotic burden.

The distribution of extracranial and intracranial atherosclerotic burden and the total CSVD score by sleep duration categories are shown in Figure S2. Compared with a normal sleep duration, a long sleep duration was correlated with an increased odds of intracranial atherosclerotic plaques (long vs. normal: OR = 1.40, 95% CI: 1.10–1.78, *p* = 0.006), but not with extracranial atherosclerotic plaques (long vs. normal: OR = 1.06, 95% CI: 0.86–1.29, *p* = 0.59). In contrast, no correlation was evident between a short sleep duration and the presence of intracranial (OR = 0.79, 95% CI: 0.57–1.09, *p* = 0.15) or extracranial atherosclerotic plaques (OR = 1.10, 95% CI: 0.87–1.39, *p* = 0.42). Of note, a similar association was observed between sleep duration and intracranial atherosclerotic burden (short vs. normal: cOR = 0.78, 95% CI, 0.57–1.08, *p* = 0.13; long vs. normal: cOR = 1.38, 95% CI, 1.09–1.75, *p* = 0.007) or extracranial atherosclerotic burden (short vs. normal: cOR = 1.14, 95% CI, 0.91–1.43, *p* = 0.27; long vs. normal: cOR = 1.07, 95% CI, 0.88–1.30, *p* = 0.49) (Figure 1). By binary or ordinal logistic regression with restricted cubic spline, subjects with a longer sleep duration displayed an increase in intracranial atherosclerotic burden and intracranial plaque presence (Figure S3).

**FIGURE 2:**
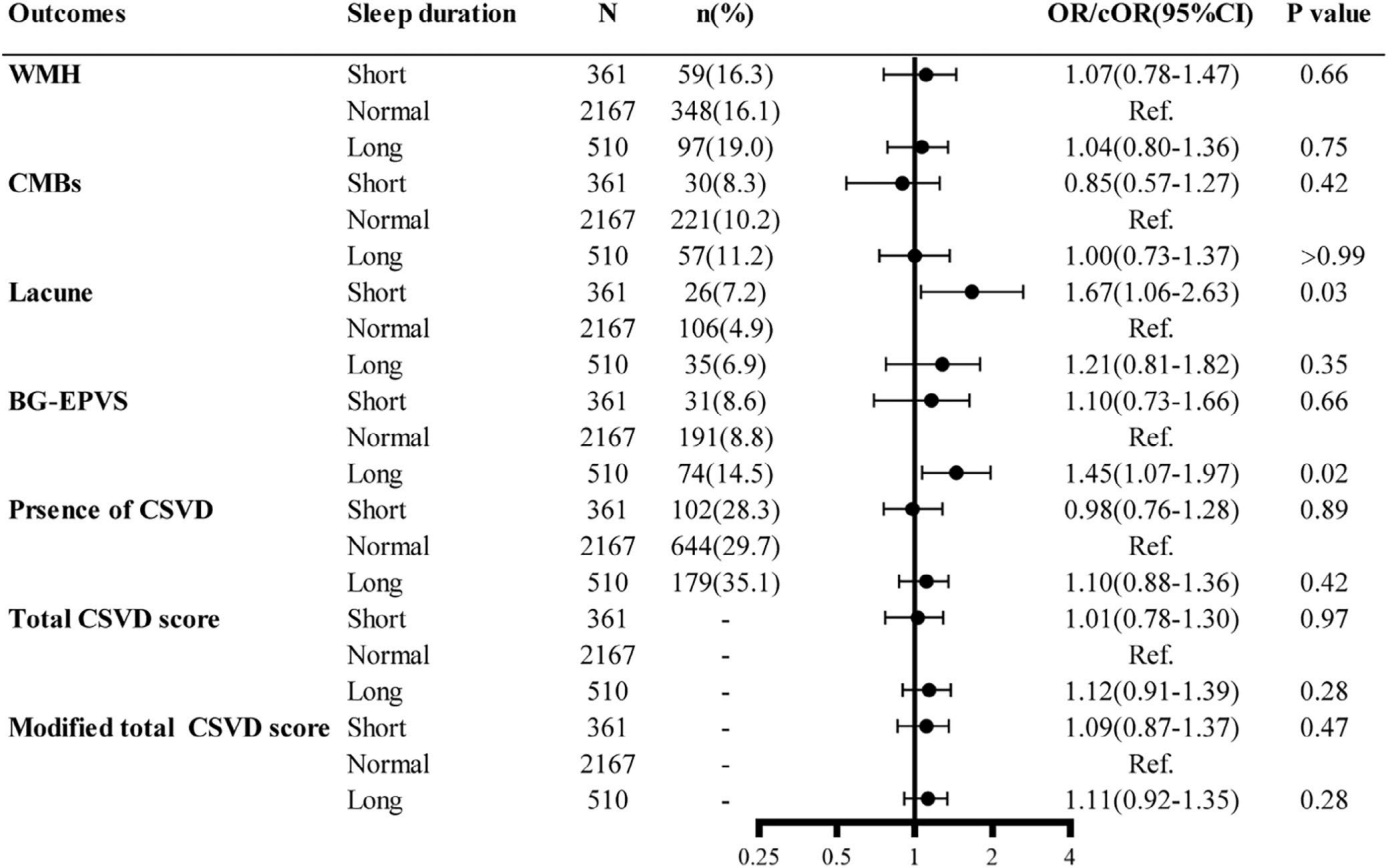
Forest plots for the association of sleep duration with CSVD imaging markers. BG‐EPVS, enlarged perivascular spaces in the basal ganglia; CI, confidence interval CSVD, cerebral small vessel disease; CMBs, cerebral microbleeds; cOR, common odds ratio; OR, odds ratio; Ref., reference; WMH, white matter hyperintensity. The degree of sleep duration was graded as short sleep (< 7 h), normal sleep (7–9 h), and long sleep (> 9 h). All models were adjusted for age, sex, body mass index, current smoking, and current drinking. The presence of WMH was defined as either confluent deep WMH (Fazekas score 2 or 3) or irregular periventricular WMH extending into the deep white matter (Fazekas score 3). The presence of lacunes and CMBs was defined as the presence of one or more lacunes or any CMB. The presence of PVS was defined as moderate to severe (grade 2–4) PVS in the basal ganglia. The presence of CSVD was defined as patient with a total CSVD score ≥ 1 point. Total CSVD scores: One point allocated to each of the following: (1) presence of lacunes, (2) presence of microbleeds, (3) moderate‐to‐severe basal ganglia PVS, and (4) severe periventricular or moderate‐to‐severe deep WMH. Modified total CSVD scores: One point allocated to the presence of lacunes, 1–4 microbleeds, frequent to severe (> 20) PVS in basal ganglia, moderate WMH (total periventricular 1 subcortical WMH grade 3–4), and 2 points allocated for ≥ 5 microbleeds and severe WMH (total periventricular + subcortical WMH grade 5–6). ORs were estimated for the presence of WMH, CMB, lacune, BG‐EPVS, and CSVD; cORs were used for total CSVD score and modified total CSVD score.

A long sleep duration was associated with BG‐EPVS (OR: 1.45, 95% CI: 1.07–1.97, *p* = 0.02) and a short sleep duration was associated with lacune (OR: 1.67, 95% CI: 1.06–2.63, *p* = 0.03) (Figure 2). However, sleep duration was not significantly correlated with WMH, CSVD, CMBs, total CSVD score, or modified total CSVD score (*p* > 0.05 for all).

### 3.3 Mediation Effect on the Association of Sleep Duration With Intracranial Atherosclerosis and CSVD Imaging Markers

**FIGURE 3:**
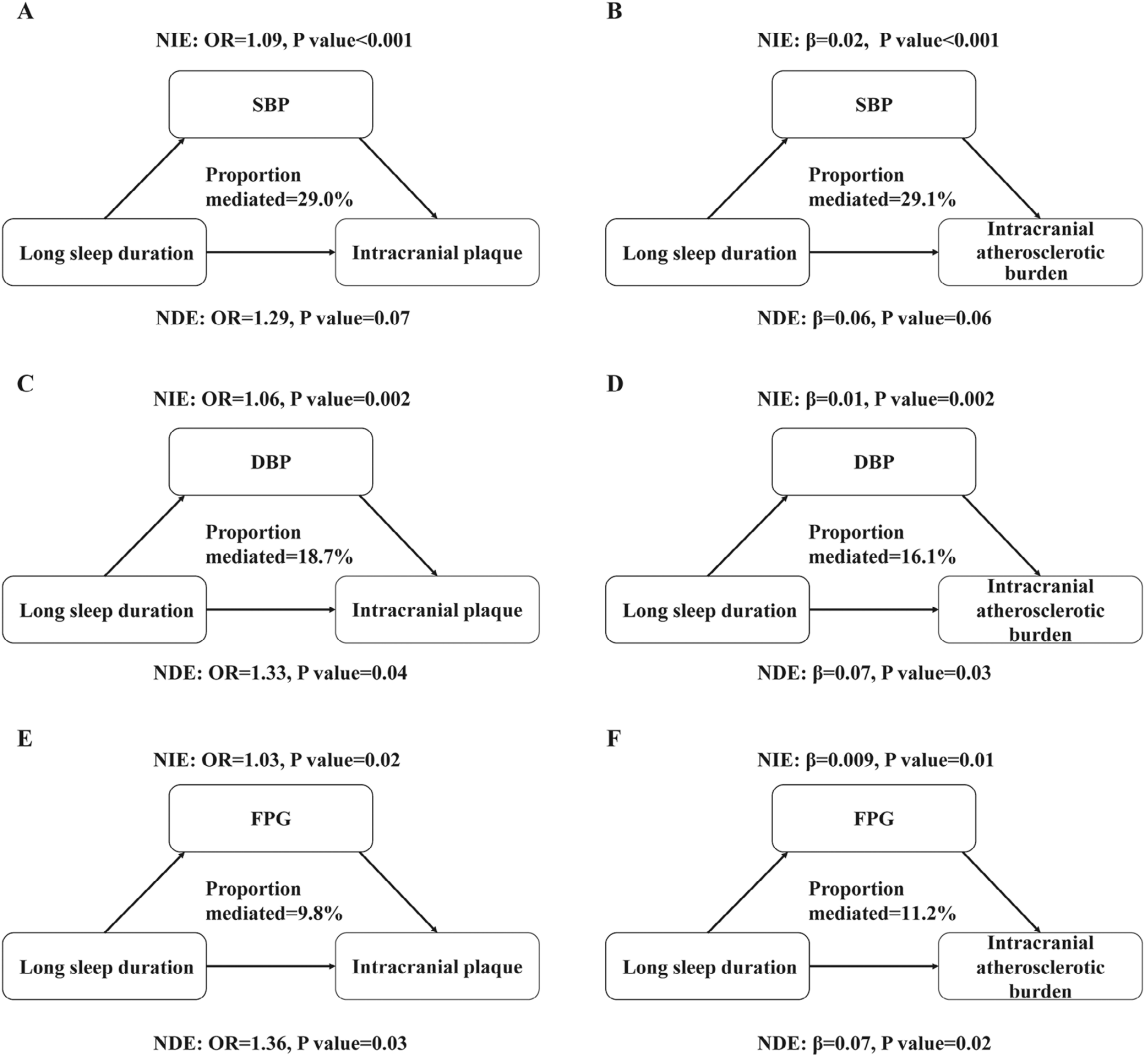
Path diagram of the mediation analysis of metabolic factors on the relationship between sleep duration and intracranial atherosclerosis. DBP, diastolic blood pressure; FPG, fasting plasma glucose; OR, odds ratio; SBP, systolic blood pressure. Long sleep duration (> 9 h) is referenced to normal sleep duration (7–9 h). The degree of intracranial atherosclerotic burden was graded as scores of 0, 1, 2–3, and ≥ 4. All models were adjusted for age, sex, body mass index, current smoking, and current drinking. ORs were estimated for the presence of intracranial plaque; β were used for intracranial atherosclerotic burden.

The analysis revealed that sleep duration was correlated with SBP, DBP, HDL‐C, and lipoprotein(a) (LP[a]) (*p* < 0.05) (Table S1); that SBP, DBP, TC, TG, LDL‐C, apolipoprotein B, and FPG were correlated with intracranial atherosclerosis (*p* < 0.05); and that SBP and DBP were associated with BG‐EPVS and lacune (*p* < 0.05) (Table S2). The partial mediating effect of metabolic factors on the correlation of a long sleep duration with intracranial atherosclerosis and intracranial atherosclerotic burden was illustrated in Figure 3 and Table 2. SBP, DBP, and FPG, respectively, accounted for 29.0%, 18.7%, and 9.8% of the correlation of sleep duration with intracranial atherosclerosis, and for 29.1%, 16.1%, and 11.2% of that between sleep duration and intracranial atherosclerotic burden. The combined mediation analysis further revealed that all mediators collectively accounted for 35.4% (95% CI: 14.1%–64.6%) of the impact of a long sleep duration on the presence of atherosclerotic plaque. Similarly, for atherosclerotic plaque burden, the combined mediating effect was 34.0% (95% CI: 12.4%–65.0%) (Figure S4).

**TABLE 2:** Mediation analysis assessing sleep duration and intracranial atherosclerosis and CSVD imaging markers through metabolic factors.

Effector	Outcome	Mediator	Total effect^c^		Natural direct effect		Natural indirect effect		Percentage mediated (%)	
			*β*/OR (95% CI)^b^	*p*	*β*/OR (95% CI)^b^	*p*	*β*/OR (95% CI)^b^	*p*	Estimate (95% CI)	*p*
Long sleep duration^a^	Intracranial atherosclerotic plaque	SBP	1.41 (1.06, 1.76)	0.02	1.29 (0.98, 1.61)	0.07	1.09 (1.04, 1.14)	< 0.001	29.0 (7.9, 50.1)	0.007
		DBP	1.41 (1.07, 1.75)	0.02	1.33 (1.01, 1.65)	0.04	1.06 (1.02, 1.09)	0.002	18.7 (3.7, 33.8)	0.01
		FPG	1.40 (1.06, 1.74)	0.02	1.36 (1.03, 1.69)	0.03	1.03 (1.01, 1.05)	0.02	9.8 (0.4, 19.2)	0.04
		Multiple mediators^d^	—	—	—	—	—	—	35.4 (14.1, 64.6)	< 0.001
	Intracranial atherosclerotic burden^f^	SBP	0.08 (0.02, 0.14)	0.01	0.06 (−0.003, 0.12)	0.06	0.02 (0.01, 0.04)	< 0.001	29.1 (4.5, 53.7)	0.02
		DBP	0.08 (0.02, 0.14)	0.01	0.07 (0.006, 0.13)	0.03	0.01 (0.004, 0.02)	0.002	16.1 (1.0, 31.2)	0.04
		FPG	0.08 (0.02, 0.14)	0.01	0.07 (0.01, 0.13)	0.02	0.009 (0.002, 0.02)	0.01	11.2 (−0.5, 22.9)	0.06
		Multiple mediators^d^	—	—	—	—	—	—	34.0 (12.4, 65.0)	< 0.001
	BG‐EPVS^h^	SBP	1.46 (1.01, 1.90)	0.046	1.38 (0.96, 1.81)	0.08	1.05 (1.02, 1.09)	0.006	16.1 (1.2, 30.9)	0.03
		DBP	1.45 (1.0003, 1.90)	0.0498	1.36 (0.94, 1.78)	0.09	1.06 (1.02, 1.11)	0.004	19.2 (1.9, 36.5)	0.03
		Multiple mediators^e^	—	—	—	—	—	—	20.3 (5.8, 51.2)	0.009
Short sleep duration^a^	Lacune^g^	SBP	1.72 (0.93, 2.51)	0.07	1.76 (0.96, 2.57)	0.06	0.98 (0.94, 1.01)	0.21	−6.0 (−16.6, 4.5)	0.26
		DBP	1.69 (0.91, 2.47)	0.08	1.75 (0.95, 2.55)	0.07	0.96 (0.92, 1.01)	0.12	−9.1 (−22.9, 4.7)	0.20

Abbreviations: BG‐EPVS, enlarged perivascular spaces in the basal ganglia; CI, confidence interval; DBP, diastolic blood pressure; FPG, fasting plasma glucose; OR, odds ratio; SBP, systolic blood pressure.

^a^ Both long sleep duration (> 9 h) and short sleep duration (< 7 h) are referenced to normal sleep duration (7–9 h).

^b^ ORs were estimated for the presence of intracranial atherosclerotic plaque, lacune, and EPVS; β were used for intracranial atherosclerotic burden.

^c^ All models were adjusted for age, sex, body mass index, current smoking, and current drinking.

^d^ Multiple mediators included SBP, DBP, and FPG.

^e^ Multiple mediators included SBP and DBP.

^f^ The degree of intracranial atherosclerotic burden was graded as scores of 0, 1, 2–3, and ≥ 4.

^g^ Presence of lacunes was defined as the presence of one or more lacunes.

^h^ Presence of PVS was defined as moderate to severe (grade 2–4) PVS in the basal ganglia.

**FIGURE 4:**
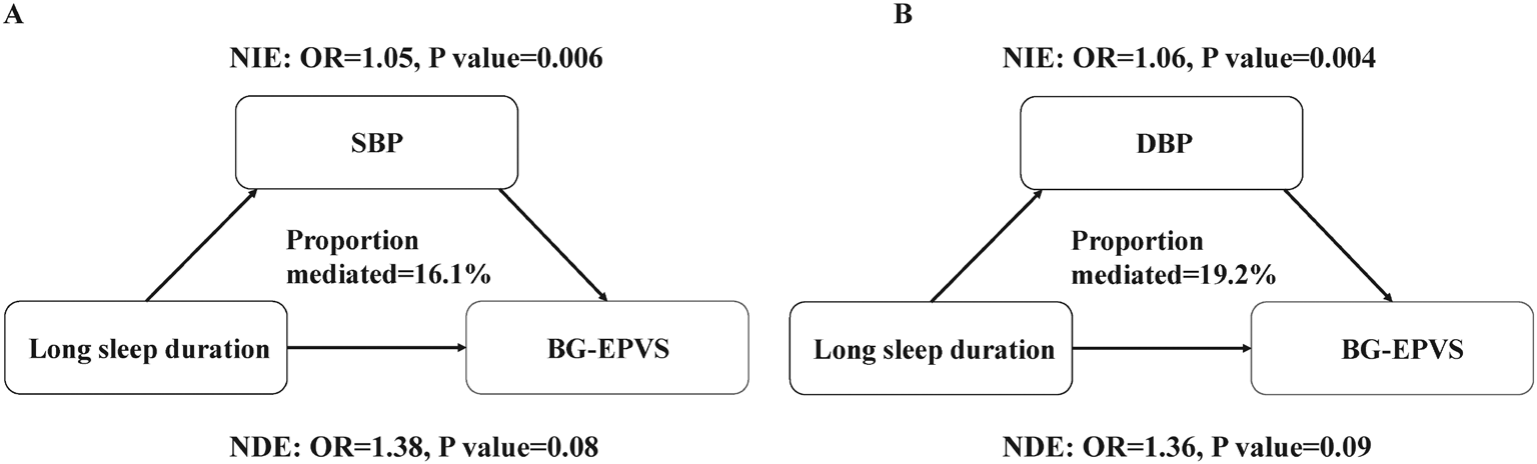
Path diagram of the mediation analysis of metabolic factors on the relationship between sleep duration and BG‐EPVS. BG‐EPVS, enlarged perivascular spaces in the basal ganglia; DBP, diastolic blood pressure; FPG, fasting plasma glucose; OR, odds ratio; SBP, systolic blood pressure. Long sleep duration (> 9 h) is referenced to normal sleep duration (7–9 h). All models were adjusted for age, sex, body mass index, current smoking, and current drinking. The presence of PVS was defined as moderate to severe (grade 2–4) PVS in the basal ganglia.

The mediating effect of metabolic factors on the correlation of a short sleep duration with lacune was non‐significant (*p* > 0.05). Moreover, SBP and DBP exerted a partial mediating effect on the correlation of a long sleep duration with BG‐EPVS by 16.1% and 19.2%, respectively (Figure 4).

## 4 Discussion

The current population‐based study demonstrated that a long sleep duration was correlated with intracranial atherosclerosis and EPVS, whereas a short sleep duration was correlated with lacune. Further mediation analysis revealed that DBP, SBP, and FPG partially mediated the effect of a long sleep duration on intracranial atherosclerosis and that SBP and DBP partially mediated that of a long sleep duration on EPVS.

Sleep duration has been documented to be implicated in atherosclerosis. Studies demonstrate that a long sleep duration is associated with carotid atherosclerosis [5, 24] and that a short sleep duration is involved in carotid atherosclerosis [4, 25]. However, in this study, a long sleep duration was correlated with intracranial atherosclerosis, but not with extracranial atherosclerosis, which was not observed for a short sleep duration, regardless of intracranial or extracranial atherosclerosis. The explanation for the observed inconsistency may lie in two aspects: first, different definitions of sleep. In Si Chen et al.'s study [4], a short sleep duration was designated as < 5 h. Second, the blood flow during sleep may change [26]. Different vascular hemodynamics are involved in carotid and intracranial arteries. Cornelia Hahn et al. suggest that blood flow plays a decisive role in the development of atherosclerosis [27] and vascular hemodynamic factors may operate differently in carotid and intracranial arteries, resulting in a distinct pathogenesis of atherosclerosis. This may result in different impacts of sleep on atherosclerosis in different parts of the arteries.

Currently, growing attention has been drawn to the link between sleep duration and the incidence of CSVD, though no definite conclusion has been reached yet. Available evidence suggests that obstructive sleep apnea is associated with CSVD imaging markers [28, 29] and that patients with ≥ 9 h of sleep often display an increased white matter hyperintensity volume (WMHV) [6]. However, controversies arise regarding the connection between sleep duration and white matter. Some studies suggest that a sleep duration of at least 7 h per night may not be associated with white matter microstructure [30]. Other studies report that a long‐term sleep fragmentation may impair the maturation of oligodendrocyte precursor cells (OPC) and decrease the integrity of the deep white matter in mice, suggesting that the blockage of OPC maturation and the pro‐inflammatory microglial activation may be implicated in the mechanisms underlying the association of sleep with white matter injury [31]. In the current study, sleep duration was not correlated with WMH, whereas a long sleep duration was correlated with EPVS and a short sleep duration was correlated with lacune, which suggests that either a long or short sleep duration may be a risk factor for CSVD.

Current evidence indicates a strong association of metabolic factors with atherosclerosis and CSVD [10, 11, 32, 33], and of sleep with metabolism [34, 35], supporting that metabolic factors may mediate the correlation of sleep with CSVD and intracranial atherosclerosis. The present study found that a long sleep duration was correlated with intracranial atherosclerosis and CSVD imaging markers. Therefore, it is arguably justified to speculate the potential involvement of metabolic factors in the findings observed. Some studies report that both a long and a short sleep duration are correlated with an elevated blood pressure [8, 36]. Other studies document that hypertension itself alters the arterial protein profile, increases the accumulation of subendothelial low‐density lipoproteins, and exacerbates the development of atherosclerosis [37] and that long‐term hypertension may induce mesangial lipid hyalinization, vessel wall thickening, and lumen narrowing of small perforating arterioles in small arterioles and molluscum arteriosum, which nourish the deeper WM [33]. Similarly, the current study also indicated blood pressure as one of the factors that mediated the impact of a long sleep duration on intracranial atherosclerosis and BG‐EPVS. Furthermore, both a short and a long sleep duration are positively correlated with type 2 diabetes [7]. Existent studies reveal that under hyperglycemic conditions, glucose accelerates the formation of atherosclerosis [38, 39] and that a diabetic condition is associated with lacunes [10, 40]. The results of our study also identified FPG as one of the factors that mediated the effect of sleep on intracranial atherosclerotic plaque and burden. Still more studies evidence that a longer sleep is correlated with lower levels of LDL, TC, and TG [9]; that triglyceride‐rich lipoproteins and LDL are risk factors for atherosclerosis and HDL is a protective factor for atherosclerosis [11]; and that LDL cholesterol is associated with larger lacunes [40]. Our study found that despite the association of a long sleep duration with HDL and LP(a) the latter two were not associated with intracranial atherosclerosis and CSVD. Collectively, these findings tentatively suggest that sleep may impact intracranial atherosclerosis through blood pressure and blood glucose, and affect BG‐EPVS through blood pressure. Future large‐scale longitudinal and experimental studies are awaited to validate the proposed mechanism.

With a comprehensive evaluation of intracranial/extracranial atherosclerotic plaques and CSVD by advanced imaging techniques, this study suggests that sleep duration is correlated with intracranial atherosclerosis and CSVD, highlighting sleep duration as a pivotal risk factor for intracranial atherosclerosis and CSVD. These findings indicate that for patients who are afflicted with poor sleep quality, close attention to their metabolic status may be beneficial for the management of intracranial atherosclerosis and CSVD.

Several limitations may remain in our study. First, sleep duration was assessed with self‐reported questionnaires, and polysomnography was not used to measure sleep duration. Second, the retrieved data only measured the sleep duration and did not record the quality of sleep and chronotype in the participants. Finally, the subject cohort consisted of the Chinese older population only. Future large‐scale investigations are needed before the results can be extrapolated to other populations.

The present study demonstrates that a long sleep duration may contribute to intracranial atherosclerosis and EPVS, while a short sleep duration may induce lacune, which may partially be mediated by SBP, DBP, and FPG. These findings highlight that for elderly people, especially those afflicted with poor sleep quality, due attention to their metabolic status may be beneficial to intracranial atherosclerosis and CSVD.

## Author Contributions

Hongbin Chen and Anqi Zhang conceived and coordinated the study; performed and executed the experiments; and wrote the manuscript. Weiqi Chen, Xueli Cai, Mengyuan Zhou, Shan Li, Jing Jing, Tiemin Wei, and Yongjun Wang carried out data collection and analysis. Yuesong Pan and Yilong Wang designed the study, double‐checked the statistical analysis, and revised the manuscript. All authors reviewed the results and approved the final version of the manuscript.

## Funding

This work was supported by the National Key R&D Program of China (nos. 2022YFC3602500, 2022YFC3602505), National Natural Science Foundation of China (no. 82425101), Beijing High‐Level Public Health Technical Personnel Construction Project (Discipline leader‐03‐12), Beijing Municipal Science & Technology Commission (no. Z231100004823036), Capital's Funds for Health Improvement and Research (2022‐2‐2045), Zhejiang provincial program for the Cultivation of High‐level Innovative Health talents and AstraZeneca Investment (China) Co. Ltd.

## Disclosure

We did not use any Artificial Intelligence Generated Content (AIGC) tools such as ChatGPT and others based on large language models (LLMs) used in developing any portion of their manuscript within the Methods section of their manuscript.

## Conflicts of Interest

The authors declare no conflicts of interest.

## Supporting information


**Data S1:** cns70797‐sup‐0001‐supinfo.docx.
**Figure S1:** Flow chart of the participant selection. PRECISE indicates Poly‐vascular Evaluation for Cognitive Impairment and Vascular Events.
**Figure S2:** Distribution of intracranial, extracranial atherosclerotic burden and CSVD score according to sleep duration. Grade 0–3 indicates intracranial and extracranial atherosclerotic burden of 0, 1, 2–3, and ≥ 4, or total CSVD score of 0, 1, 2, 3–4, respectively. The degree of sleep duration was graded as short sleep (< 7 h), normal sleep (7–9 h), and long sleep (> 9 h).
**Figure S3:** Association of sleep duration with intracranial atherosclerosis and CSVD imaging markers. BG‐EPVS, enlarged perivascular spaces in the basal ganglia; CI, confidence interval; cOR, common odds ratio; OR, odds ratio. The presence of PVS was defined as moderate to severe (grade 2–4) PVS in the basal ganglia. The presence of lacunes was defined as the presence of one or more lacunes. The reference point is the 50th percentile of sleep duration(8 h). The degree of intracranial atherosclerotic burden was graded as scores of 0, 1, 2–3, and ≥ 4. All models were adjusted for age, sex, body mass index, current smoking, and current drinking.
**Figure S4:** Parallel mediation model. DBP, diastolic blood pressure; FPG, fasting plasma glucose; SBP, systolic blood pressure. The degree of intracranial atherosclerotic burden was graded as scores of 0, 1, 2–3, and ≥ 4. Both long sleep duration (> 9 h) is referenced to normal sleep duration (7–9 h). All models were adjusted for age, sex, body mass index, current smoking, and current drinking.
**Table S1:** Association between sleep duration and metabolic factors.
**Table S2:** Association of metabolic factors with intracranial plaques and CSVD imaging markers.

## Data Availability

The data that support the findings of this study are available from the corresponding author. The data are not publicly available due to privacy or ethical restrictions.
