# Supplementary material for: Association of Sleep Duration With Intracranial Atherosclerosis and Cerebral Small Vessel Disease: A Mediation by Metabolic Factors
Source: CNS Neurosci Ther. 2026 Feb 17;32(2):e70797. doi: 10.1002/cns.70797 (PMC12910520; doi:10.1002/cns.70797)
Supplement: Supplementary file 1 — Data S1: cns70797‐sup‐0001‐supinfo.docx. Figure S1: Flow chart of the participant selection. PRECISE indicates Poly‐vascular Evaluation for Cognitive Impairment and Vascular Events. Figure S2: Distribution of intracranial, extracranial atherosclerotic burden and CSVD score according to sleep duration. Grade 0–3 indicates intracranial and extracranial atherosclerotic burden of 0, 1, 2–3, and ≥ 4, or total CSVD score of 0, 1, 2, 3–4, respectively. The degree of sleep duration was graded as short sleep (< 7 h), normal sleep (7–9 h), and long sleep (> 9 h). Figure S3: Association of sleep duration with intracranial atherosclerosis and CSVD imaging markers. BG‐EPVS, enlarged perivascular spaces in the basal ganglia; CI, confidence interval; cOR, common odds ratio; OR, odds ratio. The presence of PVS was defined as moderate to severe (grade 2–4) PVS in the basal ganglia. The presence of lacunes was defined as the presence of one or more lacunes. The reference point is the 50th percentile of sleep duration(8 h). The degree of intracranial atherosclerotic burden was graded as scores of 0, 1, 2–3, and ≥ 4. All models were adjusted for age, sex, body mass index, current smoking, and current drinking. Figure S4: Parallel mediation model. DBP, diastolic blood pressure; FPG, fasting plasma glucose; SBP, systolic blood pressure. The degree of intracranial atherosclerotic burden was graded as scores of 0, 1, 2–3, and ≥ 4. Both long sleep duration (> 9 h) is referenced to normal sleep duration (7–9 h). All models were adjusted for age, sex, body mass index, current smoking, and current drinking. Table S1: Association between sleep duration and metabolic factors. Table S2: Association of metabolic factors with intracranial plaques and CSVD imaging markers. [file CNS-32-e70797-s001.docx]

**Figure S1. Flow chart of the participant selection.** PRECISE indicates Poly-vascular Evaluation for Cognitive Impairment and Vascular Events.


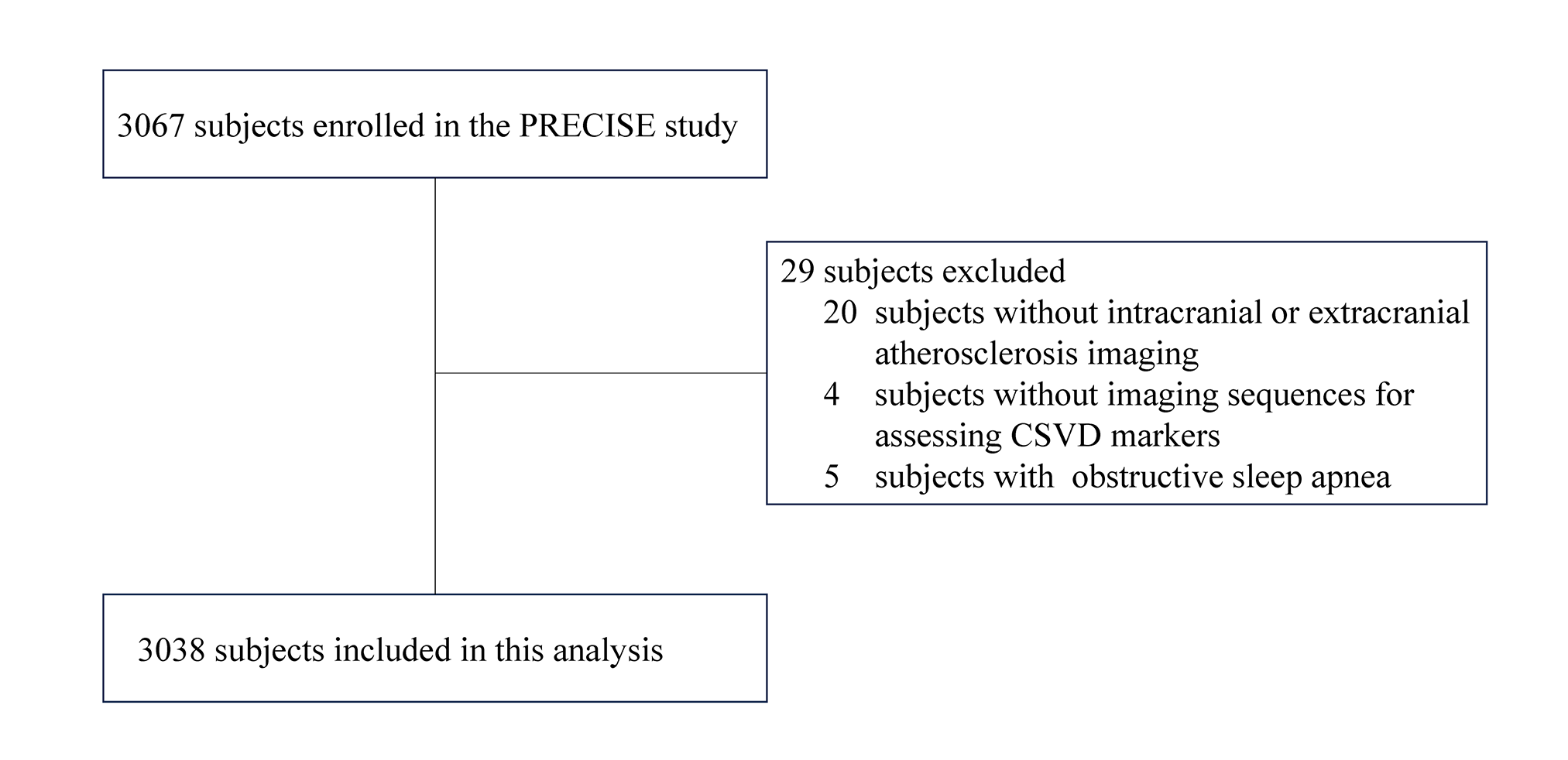


**Figure S2. Distribution of intracranial, extracranial atherosclerotic burden and CSVD score according to sleep duration.** Grade 0-3 indicates intracranial and extracranial atherosclerotic burden of 0, 1, 2-3, and ≥ 4, or total CSVD score of 0, 1, 2, 3-4, respectively. The degree of sleep duration was graded as short sleep (< 7 hours), normal sleep (7-9 hours), and long sleep (> 9 hours).

**
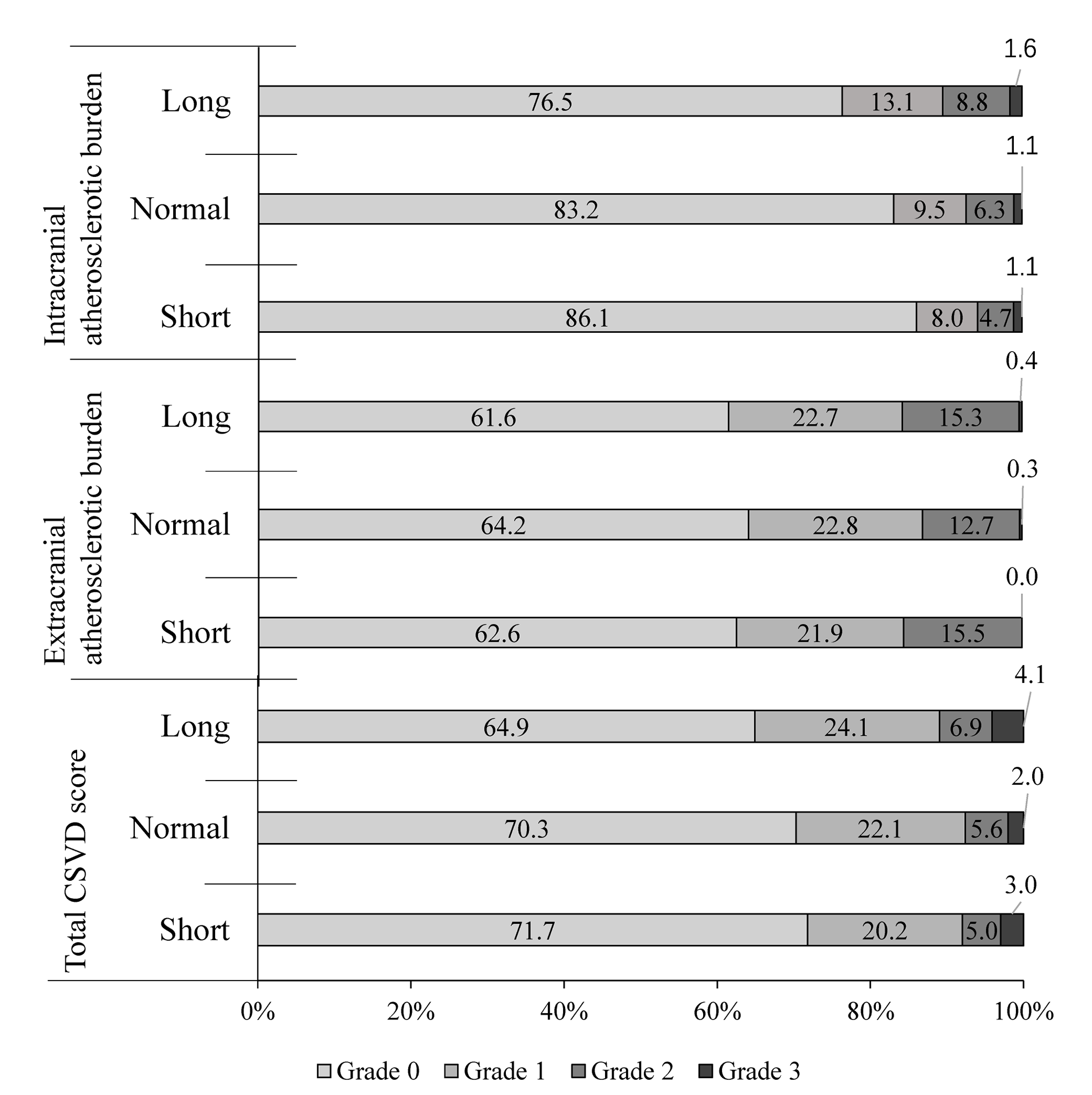
**

**Figure S3. Association of sleep duration with intracranial atherosclerosis and CSVD imaging markers.** cOR indicates common odds ratio; OR, odds ratio; CI, confidence interval; BG-EPVS, enlarged perivascular spaces in the basal ganglia. The presence of PVS was defined as moderate to severe (grade 2–4) PVS in the basal ganglia. The presence of lacunes was defined as the presence of one or more lacunes. The reference point is the 50th percentile of sleep duration(8h). The degree of intracranial atherosclerotic burden was graded as scores of 0, 1, 2–3, and ≥ 4. All models were adjusted for age, sex, body mass index, current smoking, and current drinking.


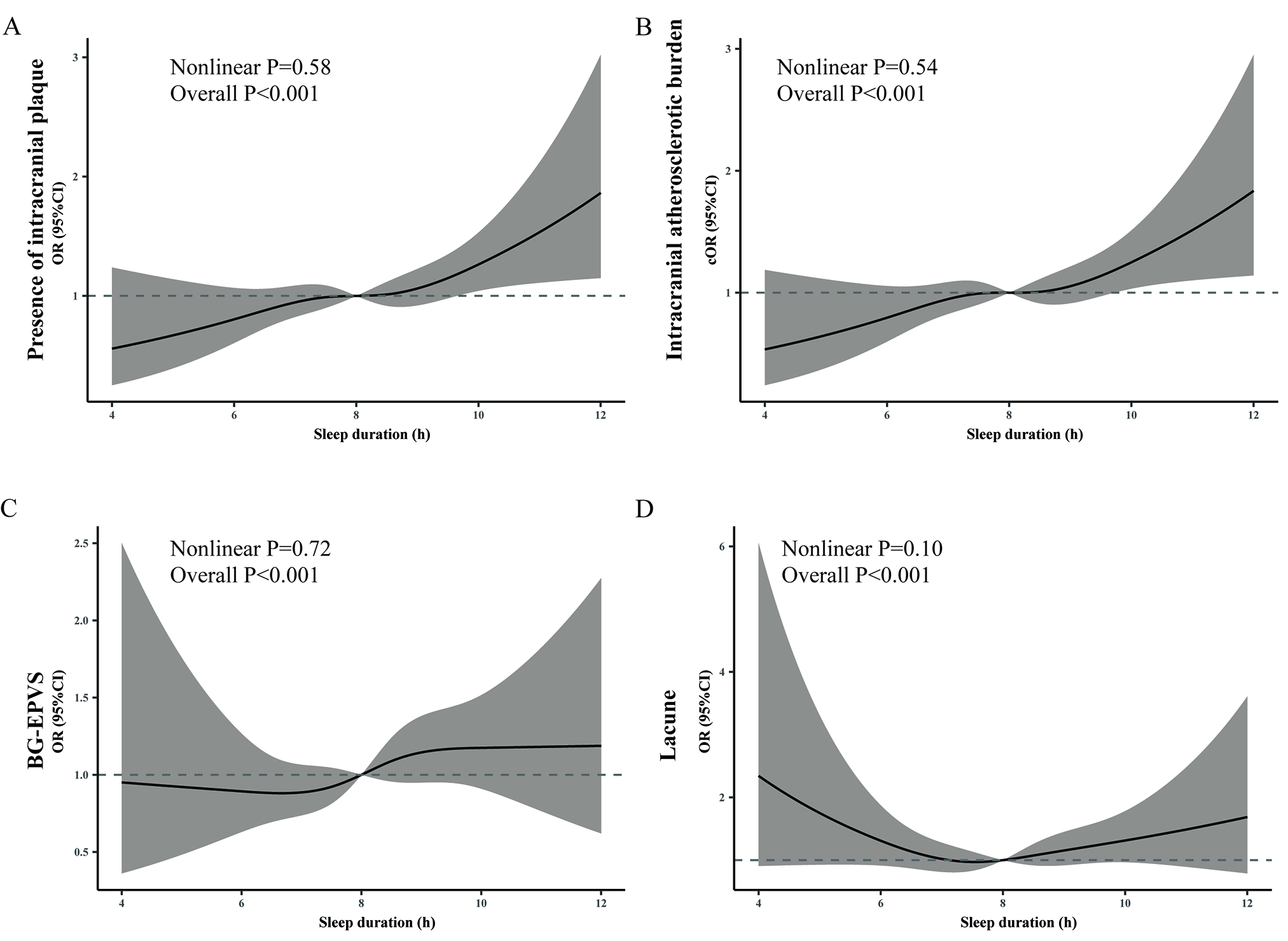


**Figure S4. Parallel mediation model.** SBP indicates systolic blood pressure; DBP, diastolic blood pressure; FPG, fasting plasma glucose. The degree of intracranial atherosclerotic burden was graded as scores of 0, 1, 2–3, and ≥4. Both long sleep duration (> 9 hours) is referenced to normal sleep duration (7-9 hours). All models were adjusted for age, sex, body mass index, current smoking, and current drinking.


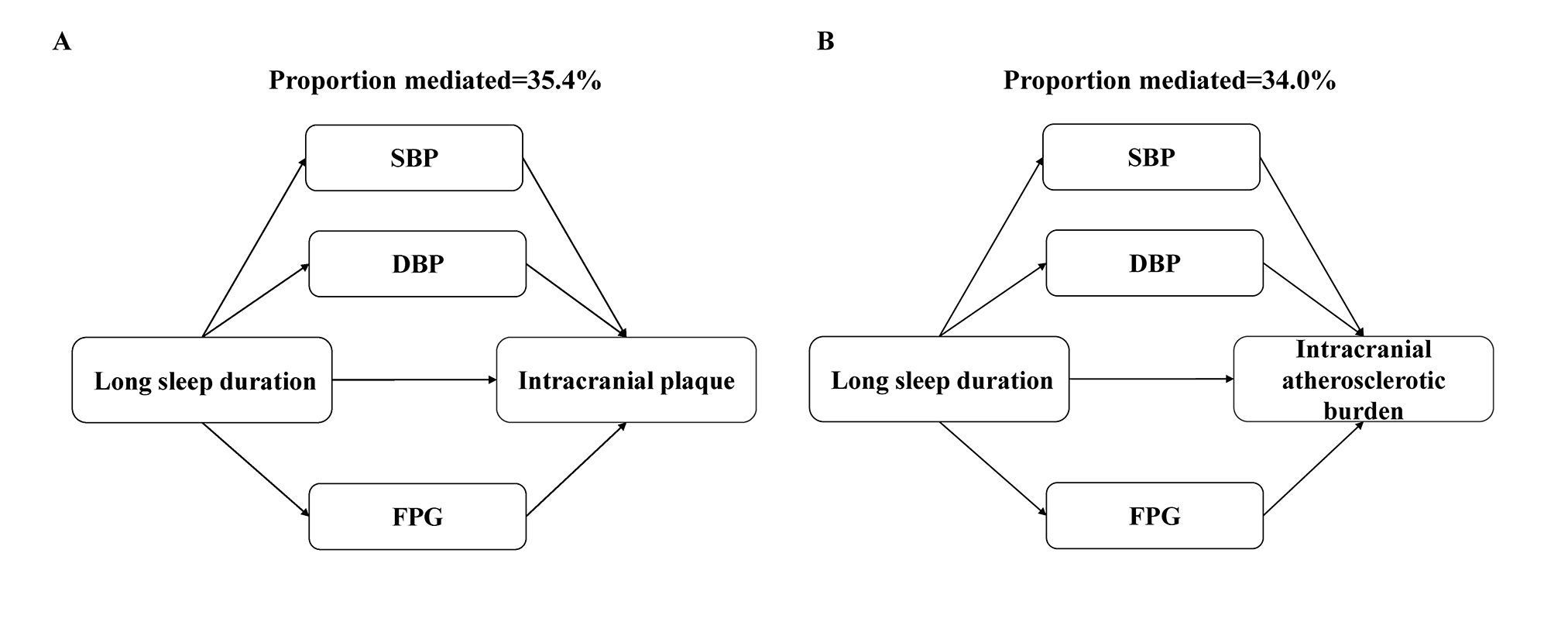


**Table S1. Association between sleep duration and metabolic factors.**

| **Outcome** | **Sleep duration**^*^ | **β**^†^ | **95%CI** | **P value** |
| --- | --- | --- | --- | --- |
| SBP | Short | -1.15 | -2.87, 0.57 | 0.19 |
|  | Normal | Ref. | Ref. |  |
|  | Long | 3.09 | 1.59, 4.58 | <0.001 |
| DBP | Short | -0.78 | -1.74, 0.19 | 0.11 |
|  | Normal | Ref. | Ref. |  |
|  | Long | 1.64 | 0.80, 2.47 | <0.001 |
| TC | Short | 0.03 | -0.08, 0.14 | 0.57 |
|  | Normal | Ref. | Ref. |  |
|  | Long | 0.07 | -0.03, 0.16 | 0.15 |
| TG | Short | -0.03 | -0.17, 0.10 | 0.63 |
|  | Normal | Ref. | Ref. |  |
|  | Long | 0.0003 | -0.12, 0.12 | 0.996 |
| HDL-C | Short | 0.009 | -0.03, 0.04 | 0.61 |
|  | Normal | Ref. | Ref. |  |
|  | Long | 0.05 | 0.02, 0.08 | <0.001 |
| LDL-C | Short | 0.05 | -0.03, 0.14 | 0.22 |
|  | Normal | Ref. | Ref. |  |
|  | Long | 0.06 | -0.01, 0.14 | 0.10 |
| LP(a) | Short | -10.79 | -30.95, 9.37 | 0.29 |
|  | Normal | Ref. | Ref. |  |
|  | Long | -20.85 | -38.34, -3.36 | 0.02 |
| ApoB | Short | 0.01 | -0.01, 0.04 | 0.30 |
|  | Normal | Ref. | Ref. |  |
|  | Long | 0.0009 | -0.02, 0.02 | 0.93 |
| FPG | Short | 0.04 | -0.13, 0.22 | 0.62 |
|  | Normal | Ref. | Ref. |  |
|  | Long | 0.20 | 0.05, 0.35 | 0.008 |

SBP indicates systolic blood pressure; DBP, diastolic blood pressure; FPG, fasting plasma glucose; TC, total cholesterol; TG, Triglyceride; HDL-C, high-density lipoprotein cholesterol; LDL-C, low-density lipoprotein cholesterol; LP(a), lipoprotein(a); Apo B, apolipoprotein B.

^*^The degree of sleep duration was graded as short sleep (< 7 hours), Normal sleep (7-9 hours), and long sleep (> 9 hours).

^†^All models were adjusted for age, sex, body mass index, current smoking, and current drinking.

**Table S2. Association of metabolic factors with intracranial plaques and CSVD imaging markers.**

| **Variables** | **Presence of intracranial plaque** | |  | **Intracranial atherosclerotic burden**^†^ | |  | **BG-EPVS**^‡^ | |  | **Lacune**^§^ | |
| --- | --- | --- | --- | --- | --- | --- | --- | --- | --- | --- | --- |
|  |  | |  |  | |  |  | |  |  | |
|  | OR (95%CI) ^*^ | P value |  | β (95%CI) | P value |  | OR (95%CI) | P value |  | OR (95%CI) | P value |
| SBP | 1.03 (1.02, 1.04) | <0.001 |  | 0.008 (0.006, 0.009) | <0.001 |  | 1.02 (1.01, 1.02) | <0.001 |  | 1.02 (1.01, 1.03) | <0.001 |
| DBP | 1.04 (1.03, 1.05) | <0.001 |  | 0.008 (0.005, 0.01) | <0.001 |  | 1.04 (1.02, 1.05) | <0.001 |  | 1.05 (1.03, 1.07) | <0.001 |
| TC | 1.20 (1.09, 1.32) | <0.001 |  | 0.04 (0.02, 0.06) | <0.001 |  | 0.91 (0.80, 1.03) | 0.14 |  | 1.05 (0.89, 1.23) | 0.57 |
| TG | 1.08 (1.01, 1.16) | 0.03 |  | 0.02 (0.004, 0.04) | 0.02 |  | 1.05 (0.95, 1.16) | 0.33 |  | 1.09 (0.98, 1.22) | 0.11 |
| HDL-C | 0.97 (0.71, 1.32) | 0.84 |  | -0.02 (-0.10, 0.05) | 0.49 |  | 0.79 (0.53, 1.19) | 0.27 |  | 0.56 (0.32, 0.97) | 0.04 |
| LDL-C | 1.16 (1.03, 1.31) | 0.01 |  | 0.03 (0.007, 0.06) | 0.01 |  | 0.93 (0.79, 1.09) | 0.36 |  | 1.12 (0.92, 1.37) | 0.26 |
| LP(a) | 1.00 (1.00, 1.001) | 0.51 |  | 0.00004 (-0.00008, 0.0002) | 0.47 |  | 1.00 (0.999, 1.001) | 0.63 |  | 1.00 (0.999, 1.001) | 0.90 |
| ApoB | 2.46 (1.62, 3.74) | <0.001 |  | 0.20 (0.10, 0.30) | <0.001 |  | 0.82 (0.46, 1.44) | 0.48 |  | 1.54 (0.77, 3.08) | 0.22 |
| FPG | 1.15 (1.09, 1.21) | <0.001 |  | 0.04 (0.03, 0.05) | <0.001 |  | 1.03 (0.97, 1.11) | 0.31 |  | 1.05 (0.98, 1.14) | 0.18 |

OR indicates odds ratio; CI, confidence interval; SBP, systolic blood pressure; DBP, diastolic blood pressure; FPG, fasting plasma glucose; TC, total cholesterol; TG, Triglyceride; HDL-C, high-density lipoprotein cholesterol; LDL-C, low-density lipoprotein cholesterol; LP(a), lipoprotein(a); Apo B, apolipoprotein B; BG-EPVS, enlarged perivascular spaces in the basal ganglia.

^*^All models were adjusted for age, sex, body mass index, current smoking, and current drinking.

^†^The degree of intracranial atherosclerotic burden was graded as scores of 0, 1, 2–3, and ≥ 4.

^‡^Presence of PVS was defined as moderate to severe (grade 2–4) PVS in the basal ganglia.

^§^Presence of lacunes was defined as the presence of one or more lacunes.
